# Clinical manifestations of vomitomania in bulimia nervosa

**DOI:** 10.1192/j.eurpsy.2021.955

**Published:** 2021-08-13

**Authors:** A. Bryukhin, E. Okonishnikova, T. Lineva, Y. Batyrev

**Affiliations:** 1 Department Of Psychiatry And Medical Psychology, RUDN University Moscow., Moscow, Russian Federation; 2 Department Of Psychiatry And Medical Psychologi, Peoples Friendship University of Russia (RUDN University), Moscow, Russian Federation

**Keywords:** eating disorder, vomitomania

## Abstract

**Introduction:**

In the dynamics of bulimia nervosa, a significant proportion of patients show a pathological attraction to purifying behavior in the form of artificially induced vomiting. This variant of the pathology of the drives significantly aggravates the symptoms, causes a severe degree of maladaptation of patients and great difficulties in the treatment of the disease.

**Objectives:**

To identify and describe the manifestations of vomitomania in patients with bulimia nervosa, the impact on the outcome of the disease.

**Methods:**

Clinico-psychopathological, psychological, catamnestic.

**Results:**

120 patients with bulimia nervosa were examined: 112 women and 8 men aged 22-43 years. 86 of them (80 - women, 6 - men) were found to have vomitomania (a pathological urge to induce vomiting). Patients with pleasure, without feeling shy, awkward, described their own vomiting behavior - noted the expectation of vomiting, prepared for its implementation, observing complex rituals, imagined the vomiting act and its consequences in their minds, imagination. Describing vomiting, patients used superlative degrees of comparison, noted a sense of bliss, pleasure, “high” in the implementation of this irresistible desire. If it was impossible to induce vomiting, there was a feeling of depression, depressed mood, irritability, anger, physical distress - in fact, manifestations of withdrawal syndrome. Critical attitude to the above-described pathological behavior was absent in a significant part of cases.

**Conclusions:**

Special pathological attraction to vomiting – vomitomania - is a widespread symptom of bulimia nervosa and drive disorders in this disease. It presents significant challenges for therapy especially in comorbid bulimia nervosa with personality disorders and schizotypal disorder.

